# Cloning and Functional Characterization of NADPH-Cytochrome P450 Reductases in *Aconitum vilmorinianum*

**DOI:** 10.3390/molecules28217409

**Published:** 2023-11-03

**Authors:** Jingping Cheng, Guodong Li, Xue Wang, Congwei Yang, Furong Xu, Zigang Qian, Xiaohui Ma

**Affiliations:** 1 College of Chinese Materia Medica, Yunnan Key Laboratory of Southern Medicinal Utilization, Yunnan University of Chinese Medicine, Kunming 650500, China; 2 Key Laboratory of Yunnan Provincial Department of Education on Substance Benchmark Research of Ethnic Medicines, Kunming 650500, China

**Keywords:** *Aconitum vilmorinianum*, NADPH-cytochrome P450 reductase, diterpene alkaloids, class II CPR, transfer electrons

## Abstract

Diterpenoid alkaloids (DAs) are major pharmacologically active ingredients of *Aconitum vilmorinianum*, an important medicinal plant. Cytochrome P450 monooxygenases (P450s) are involved in the DA biosynthetic pathway, and the electron transfer reaction of NADPH-cytochrome P450 reductase (CPR) with P450 is the rate-limiting step of the P450 redox reaction. Here, we identified and characterized two homologs of CPR from *Aconitum vilmorinianum*. The open reading frames of *AvCPR1* and *AvCPR2* were found to be 2103 and 2100 bp, encoding 700 and 699 amino acid residues, respectively. Phylogenetic analysis characterized both *AvCPR1* and *AvCPR2* as class II CPRs. Cytochrome *c* and ferricyanide could be reduced with the recombinant proteins of AvCPR1 and AvCPR2. Both *AvCPR1* and *AvCPR2* were expressed in the roots, stems, leaves, and flowers of *A. vilmorinianum*. The expression levels of *AvCPR1* and *AvCPR2* were significantly increased in response to methyl jasmonate (MeJA) treatment. The yeasts co-expressing *AvCPR1*/*AvCPR2*/*SmCPR1* and *CYP76AH1* all produced ferruginol, indicating that AvCPR1 and AvCPR2 can transfer electrons to CYP76AH1 in the same manner as SmCPR1. Docking analysis confirmed the experimentally deduced functional activities of *AvCPR1* and *AvCPR2* for FMN, FAD, and NADPH. The functional characterization of *AvCPR*s will be helpful in disclosing molecular mechanisms relating to the biosynthesis of diterpene alkaloids in *A. vilmorinianum*.

## 1. Introduction

*Aconitum vilmorinianum* Kom. is an important medicinal plant and is mainly distributed in southwest China [1]. As a main raw material of Yunnan Baiyao, the root of *A. vilmorinianum* is widely used in the treatment of trauma-associated injuries. Diterpenoid alkaloids (DAs) are major pharmacologically active ingredients of *A. vilmorinianum*, with a variety of biological activities, such as analgesic, anticancer, and cardiac activities [2]. To date, more than 40 diterpenoid alkaloids have been isolated from *A. vilmorinianum*, including yunaconitine, vilmorrianine A, and vilmorrianine B [3]. However, the content of DAs in *A. vilmorinianum* is only 0.1–0.5%, and the complex structure makes it difficult to largely obtain them via chemical synthesis or semisynthesis [4]. Therefore, it is important to analyze the biosynthetic pathways of DAs in *A. vilmorinianum* to improve the active ingredients via genetic engineering or to realize the heterologous production of diterpene alkaloids via synthetic biology. According to the structures of DAs, cytochrome P450 monooxygenases (P450s) are closely involved in their biosynthetic pathway.

The P450 family is the largest gene family in plants [5]. P450s are involved in the biosynthesis of many natural products, producing a vast diversity of compounds. Plant P450s can catalyze a wide variety of reactions in secondary metabolism, including hydroxylation, reduction, decarboxylation, sulfoxidation, N- and O-demethylation, epoxidation, deamination, and dehalogenation [6]. Almost all eukaryotic P450s belong to class II microsomal P450 systems, which are composed of P450s and NADPH-cytochrome P450 reductases (CPRs) [7]. The catalytic activities of most eukaryotic P450s depend on their reduction partner CPRs. CPRs transfer two electrons from NADPH through the flavin adenine dinucleotide (FAD) and flavin mononucleotide (FMN) cofactors into the heme iron center of P450s (http://www.p450.kvl.dk/p450rel.shtml, accessed on 29 July 2022). CPR, containing an N-terminally positioned FMN-binding domain and a C-terminally positioned NADPH/FAD-binding domain, belongs to a small family of diflavin proteins [7]. Angiosperm CPRs are generally classified into two classes, class I and class II, according to the sequences of the N-terminus to the hydrophobic region [8]. CPR-I generally has a shorter stretch of residues in the N-terminal protein sequence preceding the membrane-spanning domain than CPR-II [8,9]. The number of CPR isoforms in plants varies from one to four depending on the species [8,10,11,12,13]. The function of CPRs with P450s in plants has been studied in several species [14,15,16,17]. Because of the different electrostatic potentials of CPR and P450, the interaction efficiency of CPR-P450 may be somewhat modulated depending on the CPR homolog present [7]. The pairing of P450 and different CPRs may result in different productivities of plant-specific metabolites in heterologous systems. Six CPRs from different plants were inserted together with *Uni25647* and *CYP72A154* into the yeast genome to examine the coupling efficiency of P450 with different CPRs [18]. CPR from *Glycyrrhiza uralensis* (GuCPR1) showed the highest coupling efficiency with *Uni25647* and *CYP72A154* [18]. Microsomes from SF9 insect cells, with a combination of the P450 enzyme geraniol 10-hydroxylase (*G10H* from *Catharanthus roseus)* and *NfCPR2* (*Nothapodytes foetida*), showed a higher conversion rate of naringenin to eriodictyol than those with a combination of *G10H* and *AtCPR1* (*Arabidopsis thaliana*) [17]. CPRs play indispensable roles in the biosynthesis of plant secondary metabolites. Numerous plant CPRs are beneficial for the production of secondary metabolites, which provide multiple options to improve the efficiency of metabolic engineering in heterologous systems.

In this study, we describe the isolation and characterization of cDNAs encoding two CPR isoforms from *A. vilmorinianum*. The expression levels of two CPR isoforms in different organs and methyl jasmonate (MeJA)-treated *A. vilmorinianum* were examined. Furthermore, the two CPR isoforms were co-expressed in yeast with *CYP76AH1,* and their function was elucidated. The electron transfer reaction of CPR with P450 is the rate-limiting step of the P450 redox reaction. Therefore, the cloning and functional study of the CPR gene are important for the biosynthesis of DAs in *A. vilmorinianum* and can help to advance the biosynthesis of the diterpenoid alkaloid components of plants.

## 2. Results

### 2.1. Cloning and Analysis of AvCPRs

**Figure 1:**
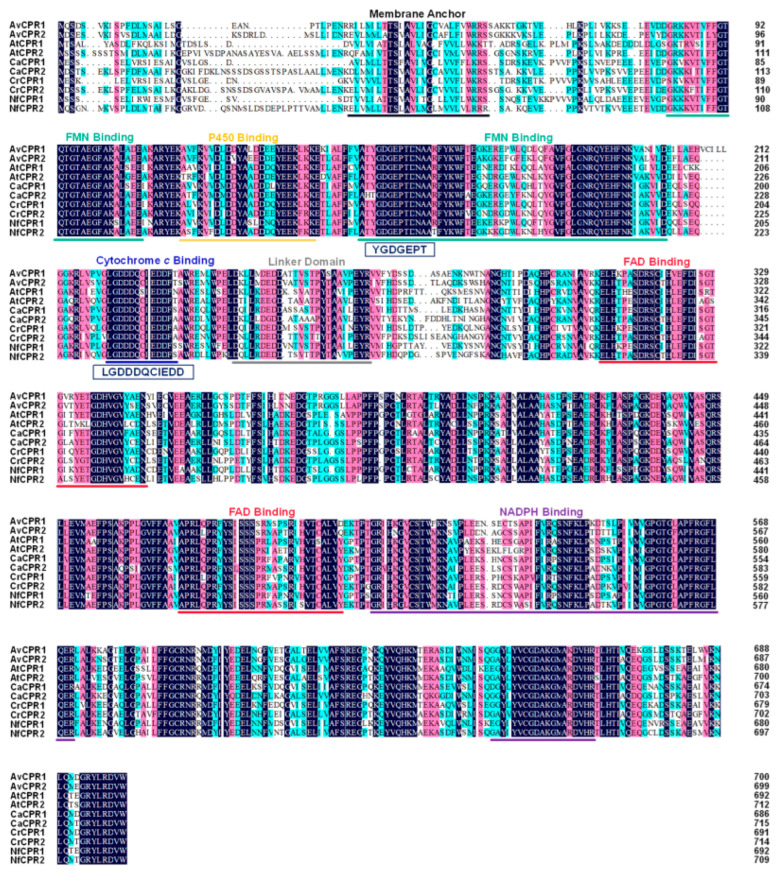
Multiple sequence alignment of the characterized plant CPRs and AvCPRs using DNAMAN. Conserved domains are underlined as follows, transmembrane region, FMN-binding domain, P450-binding domain, cytochrome *c*-binding domain, FAD-binding domain, NADPH-binding domain, and linker domain.

A total of six genes were found in the transcriptome of *A. vilmorinianum*. After comparison and annotation analysis was performed on the Basic Local Alignment Search Tool (BLAST) (http://www.ncbi.nlm.nih.gov/BLAST, accessed on 29 May 2020), two genes were finally annotated as CPRs. Two *CPR* genes in *A. vilmorinianum*, named *AvCPR1* (GenBank accession no. OR037125) and *AvCPR2* (GenBank accession no. OR037126), were amplified with the specific primers listed in Table S1. The ORFs of *AvCPR1* and *AvCPR2* were found to be 2103 bp and 2100 bp, coding proteins of 700 and 699 amino acids, respectively. AvCPR1 was 78.42% identical to AvCPR2. The physical and chemical properties and secondary structures of AvCPRs are shown in Tables S2 and S3. The results predicted by TMHMM showed that both AvCPR1 and AvCPR2 had transmembrane regions at the N-terminus (Figure S1). Amino acid sequence alignment showed that both AvCPR1 and AvCPR2 identified closely with CPRs from other plants (Figure 1). Several conserved functional domains of CPRs were identified in AvCPRs. The FAD-binding domain, FNM-binding domain, P450-binding domain, cytochrome *c*-binding domain, and NADPH-binding domain were evolutionarily conserved in CPR. The N-terminal membrane-anchoring region, which may be required for its location on the endoplasmic reticulum, showed high differentiation. Tryptophan is the last C-terminal amino acid residue of most plant CPRs, located in the nicotinamide-binding site of CPRs, which regulates the binding and release of NADPH [19].

**Figure 2:**
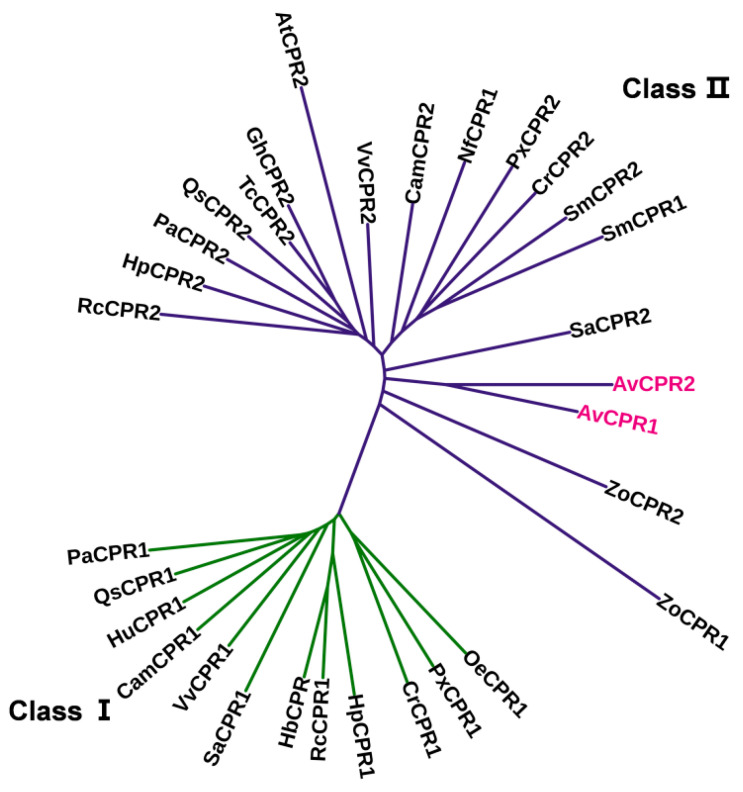
Neighbor-joining phylogenetic tree of CPRs from 17 plant species. Multiple alignments were conducted using the ClustalW program, and the tree was constructed with MEGA 6.06. The bootstrap values were generated after 1000 replicates.

The phylogenetic tree for CPR proteins in *A. vilmorinianum* and other plants was constructed using the MEGA 6 program with the neighbor-joining algorithm (Figure 2). The phylogenetic analysis of CPRs showed that plant CPRs were clustered into two groups, named class I and class II. Both AvCPR1 and AvCPR2 were grouped in class II.

### 2.2. Catalytic Activities of Recombinant AvCPRs

**Figure 3:**
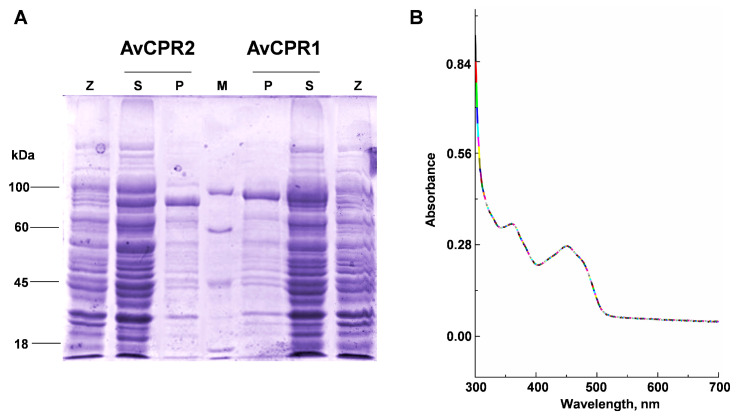
SDS-PAGE analyses and UV spectrum of the recombinant proteins of AvCPR1 and AvCPR2. (**A**) SDS-PAGE analyses of the recombinant proteins of AvCPRs. Lane M: molecular weight marker; Lane Z: pET-32a in BL21 (DE3); Lane S: supernatant proteins; Lane P: purified proteins. (**B**) UV spectrum of recombinant AvCPRs.

To confirm the functions of AvCPRs, *AvCPR1* and *AvCPR2* were heterologously expressed in BL21 (DE3). To increase the solubility of AvCPRs, 51 and 55 amino acids, the N-terminal membrane-anchoring regions of AvCPR1 and AvCPR2, respectively, were truncated. SDS-polyacrylamide gel electrophoresis (SDS-PAGE) results showed that the truncated AvCPR1 and AvCPR2 were expressed in soluble forms after induction with IPTG for 12 h (Figure 3A). Similar to other plant CPRs, the recombinant protein of AvCPRs showed characteristic absorbance bands of a flavoprotein with prominent peaks at 450 and 380 nm (Figure 3B).

**Figure 4:**
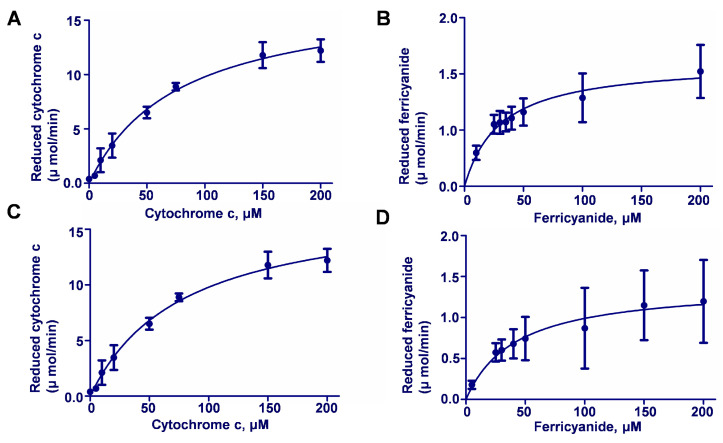
Enzyme activity assay of recombinant proteins of AvCPRs. (**A**) The kinetic parameters of AvCPR1 for cytochrome *c*. (**B**) The kinetic parameters of AvCPR1 for K_3_Fe(CN)_6_. (**C**) The kinetic parameters of AvCPR2 for cytochrome *c*. (**D**) The kinetic parameters of AvCPR2 for K_3_Fe(CN)_6_. Values are means ± SDs (n = 3).

An enzyme activity assay in vitro showed that AvCPR1 and AvCPR2 activities for cytochrome *c* and K_3_Fe(CN)_6_ followed typical Michaelis–Menten curves (Figure 4). The steady-state kinetic constants of AvCPR1 and AvCPR2 for cytochrome *c* and K_3_Fe(CN)_6_ were determined (Table 1). AvCPR2 had a higher affinity for cytochrome *c* than AvCPR1. AvCPR1 and AvCPR2 showed similar affinities for K_3_Fe(CN)_6_. Compared to cytochrome *c*, AvCPR1 and AvCPR2 were preferentially combined with K_3_Fe(CN)_6_ with lower *K_m_* values. On all accounts, both cytochrome *c* and K_3_Fe(CN)_6_ were reduced by AvCPRs in an NADPH-dependent manner. Thus, both AvCPR1 and AvCPR2 appeared functionally active as CPR.

### 2.3. AvCPR1 and AvCPR2 Supported Heterogeneous P450 Monooxygenase Activity

**Figure 5:**
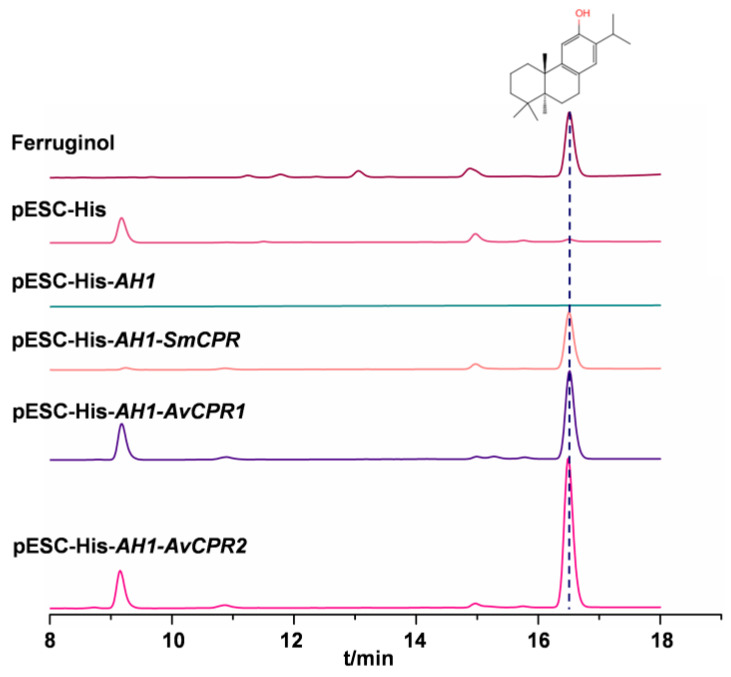
HPLC analysis of yeast products.

The ORFs of *AvCPRs*, *SmCPR*, and *CYP76AH1* were inserted into the yeast epitope-tagging vector pESC-His. The miltiradiene-producing yeast strain YJ14 was used as the chassis strain [20]. The transgenic strains co-expressing *AvCPRs* or SmCPR with *CYP76AH1* produced ferruginol (Figure 5). In contrast, ferruginol was not detected in the extracts of the transgenic strains expressing empty vector pESC-His or pESC-His-*AH1*. Both *AvCPR1* and *AvCPR2* could support CYP76AH1 catalytic activity similar to that with *SmCPR*, indicating that *AvCPR1* and *AvCPR2* were functional NADPH-cytochrome P450 reductases that facilitate P450 activity in vivo.

### 2.4. Expression Profiles of AvCPRs in A. vilmorinianum

**Figure 6:**
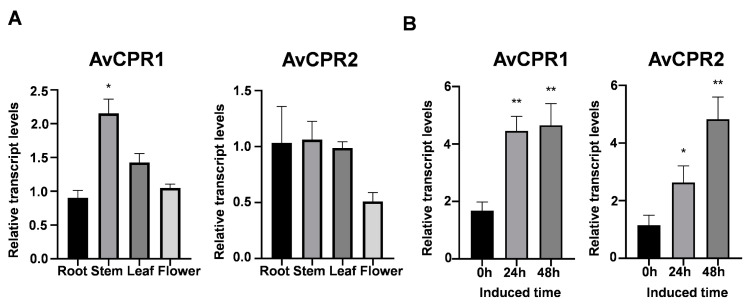
Expression of *AvCPRs in A. vilmorinianum*. (**A**) Expression patterns of AvCPRs in different organs. (**B**) Expression levels of AvCPRs in leaves with MeJA treatment. Values are means ± SDs (n = 3) (* *p* < 0.05; ** *p* < 0.01).

The results of mRNA expression patterns showed that *AvCPRs* were expressed in all tissues of *A. vilmorinianum*, indicating that *AvCPRs* were constitutively expressed in *A. vilmorinianum* to support P450 oxidation reactions (Figure 6A). Diterpenoid alkaloids were found to be distributed in different tissues of *A. vilmorinianum* [21]. The expression of *AvCPRs* showed similar expression levels with the distribution of diterpenoid alkaloids in all examined tissues of *A. vilmorinianum*, indicating that both *AvCPR1* and *AvCPR2* may be associated with the biosynthesis of diterpenoid alkaloids. Methyl jasmonate (MeJA) is a commonly used abiotic elicitor; in some cases, a simultaneous induction of compounds and CPRs has been detected. In *Andrographis paniculata*, *ApCPR2* was inducible using MeJA and its pattern matched with andrographolide accumulation [11]. MeJA induces an increase in the transcript levels of *WsCPR2* and the content of two key withanolides in *Withania somnifera* (L.) Dunal [16]. We explored the induction of AvCPRs using MeJA in *A. vilmorinianum.* The results showed that the expression of *AvCPR1* and *AvCPR2* increased significantly from 0 to 48 h after being treated with MeJA, indicating that both *AvCPR1* and *AvCPR2* were induced by MeJA (Figure 6B).

### 2.5. Docking Analysis of AvCPR1 and AvCPR2

*Arabidopsis* ATR2 (PDB ID: 5GXU) is derived from the model plant *Arabidopsis thaliana*, whose protein structure has been obtained via crystallization. Based on the crystal structure of ATR2, the 3-D structures of AvCPRs were predicted and constructed using Phyre 2 (Figure S2A,B). Sequence alignment results show that AvCPR1 and AvCPR2 shared a 79% identity score with ATR2. Analyses of the evolutionary conservation of AvCPR surface amino acids indicated that many amino acid residues were conserved in CPRs (Figure S2C,D). The highly conserved amino acid residues were found to be functional and structural residues of AvCPRs through the bioinformatics tools of the ConSurf server.

**Figure 7:**
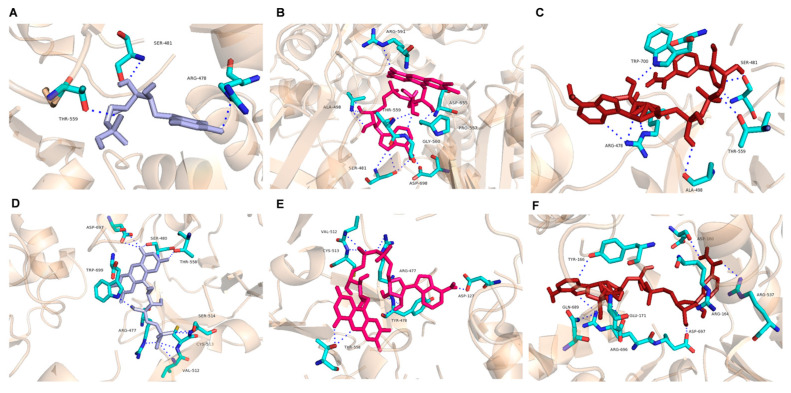
Docking sites of modeled AvCPRs with FMN, FAD, and NADPH. (**A**) AvCPR1 with FMN. (**B**) AvCPR1 with FAD. (**C**) AvCPR1 with NADPH. (**D**) AvCPR2 with FMN. (**E**) AvCPR2 with FAD. (**F**) AvCPR2 with NADPH. The active binding sites were visualized in PyMol 2.0 software.

The binding energies of AvCPR1 to FMN, FAD, and NADPH were −9.2, −10.4, and −8.7 kcal mol^−1^, respectively. The binding energies of AvCPR2 to FMN, FAD, and NADPH were −9.6, −9.7, and −10.0 kcal mol^−1^, respectively. The binding energies indicated that both AvCPR1 and AvCPR2 had fine interactions with ligands of NADPH, FAD, and FMN with different spatial conformations (Figure S3). AvCPR1 and AvCPR2 had different ligand sites (Figure 7), and the residues that play important roles in ligand binding were almost all conserved residues within AvCPRs. The electrostatic energy on the surfaces of AvCPRs binding to FMN and FAD showed that an acidic patch in the FMN domain and a basic patch in the FAD domain were involved in the interaction (Figure S4).

## 3. Discussion

The plant cytochrome P450 gene family is the largest gene family in plants [5] (Nelson and Werck-Reichhart 2011). However, as a redox partner for P450, the number of CPR genes was relatively small. The estimated ratio between CPR and P450 in the microsomal membrane is 1:15 [22]. The high ratio of P450 to CPR implies that P450s compete for CPRs, and the electron transport between CPR and P450 must be particularly organized to ensure electron supply from CPRs to large numbers of P450s at once [22]. Yeast and mammals only harbor one CPR [23,24]. In contrast, plants may contain one to four CPR paralogous genes [8,10,11,12,13]. A single CPR has been characterized from some plants, such as *Coleus blumei*, *Papaver somniferum*, and *Vigna radiata* [25,26,27]. *Capsicum annuum*, *Arabidopsis thaliana*, *Camptotheca acuminata*, *Petroselinum crispum*, and *Siraitia grosvenorii* have two CPR homologs [12,14,28,29,30]. *Nothapodytes foetida* and *hybrid poplar* (*Populus trichocarpa* × *Populus deltoides*) contain three CPR paralogs [8,17]. Four CPR paralogs were identified from *Andrographis paniculate* [10,11]. In this study, two paralogs of CPR from *A. vilmorinianum* were cloned and characterized. Phylogenetic analysis showed that both AvCPR1 and AvCPR2 were grouped into class II. CPR class II is believed to play a more important role in defense and adaptation mechanisms than CPR-I [9,16]. This is supported by the increased expressions of AvCPR1 and AvCPR2 in response to MeJA treatment. A previous study on *Withania somnifera* (L.) Dunal found that treatment with MeJA induced the expression of WsCPR2, which positively affected the accumulation of withanolides [16]. *AvCPRs* may participate in the secondary metabolism of *A. vilmorinianum* and may be involved in the biosynthesis of DAs.

CPRs transfer electrons from NADPH through the FAD and FMN cofactors into the central heme iron of P450s, and the FMN domain is the domain that interacts with P450. Sequence alignments demonstrated that the AvCPRs consisted of a membrane anchor, an FMN-binding domain in the N-terminal region, a linker domain, and a C-terminally positioned FAD- and NADPH-binding domain. Acidic residues (-LGDDDQCIEDD/-YGDGEPT), which are thought to be important in CPR, P450 interactions, and highly conserved negatively charged residues (in plants, animals, and fungi), also exist in AvCPRs [7,31,32]. These amino acid sequences are thought to interact with cytochrome *c* and P450 [33,34]. The linker domain between FMN- and FAD-binding domains (Figure 1) has an important function as a flexible hinge. It is responsible for bringing the two flavin domains in close proximity to facilitate internal electron transfer within CPR and allow CPR to interact with P450 [34,35]. Both P450 and CPRs are attached to the endoplasmic reticulum using an N-terminal “anchor” region [36]. Based on the sequence alignments, AvCPRs exhibit the greatest variation in membrane-anchored N-terminal sequences due to the low selection pressure to preserve specific residues within this region, suggesting that the role of the N-terminal structural domain is membrane-anchored rather than being subject to catalytic activity. Further predictions of AvCPRs using docking showed high affinities with FMN, FAD, and NADPH. The residues that play important roles in ligand binding were found within AvCPRs. To study the functions of these residues, site-directed mutagenesis experiments need to be performed in future work. Interaction between CPR and P450s is thought to be based on electrostatic interactions between a negatively charged region near the bound FMN cofactor and a positively charged indentation near the heme of the P450 enzyme [37]. The electrostatic energy on the surfaces of AvCPRs via docking also found negative charge near the FMN factor. Afterwards, related experiments need to be conducted in future work to analyze the role of AvCPRs with P450s.

Functional plant CPRs can serve as partners to various P450s. However, the catalytic activity of P450 varies with the sources and amount of CPRs. Three CPRs were chosen to evaluate the hydroxylation activity of *F3*′*H*, and *F3*′*H* coupled with *CrCPR* (*Catharanthus roseus*) led to the highest amount of hydroxylation production. The conversion ratio of naringenin to eriodictyol and taxifolin catalyzed by *F3*′*H* reached 90.5% and 56.8% by increasing the gene copy number of *CrCPR* to optimize the ratio of *F3*′*H*-*CPR* to 1:2 [38]. Co-expressing different combinations of *CYP716As* and *CYP72As* with different CPR classes from three legumes (*Medicago truncatula*, *Lotus japonicus*, and *Glycyrrhiza uralensis*) in transgenic yeast indicated that *CYP716As* worked better with CPR-I from the same species, while *CYP72As* worked better with CPR-IIs. *CYP88D6* paired with class II *GuCPR* (*Glycyrrhiza uralensis*) produced the highest level of 11-oxo-β-amyrin in engineered yeast strains [39]. *AvCPRs* could support the catalytic activity of *CYP76AH1*, indicating that both *AvCPR1* and *AvCPR2* were functional NADPH-cytochrome P450 reductases. As the reduction partner of P450, CPRs are widely used in metabolic engineering for the production of plant secondary metabolites in heterologous systems. The interaction efficiency of CPR-P450 can be raised by selecting CPR genes from different species or optimizing the ratio of P450s to CPRs. In this study, we provided two new plant CPR elements, *AvCPR1* and *AvCPR2*, indicating a new perspective centered around the limitations of the medicinal production application of *A. vilmorinianum*, which laid the foundation for the biosynthesis of diterpene alkaloids in *A. vilmorinianum* and provided a new option for improving the catalytic activity of P450. Later, we will investigate the P450s related to the biosynthesis of DAs, an important active ingredient in *A. vilmorinianum*. Different combinations of AvCPRs with P450s will be performed to determine the best pairing to improve the heterologous production of DAs.

## 4. Materials and Methods

### 4.1. Plant Materials

*A. vilmorinianum* was collected from Liang Wang Mountain, Chengjiang County, Yunnan Province, China, and cultured in the culture room of Yunnan University of Chinese Medicine. The *A. vilmorinianum* samples were harvested after being treated with 100 μM MeJA for 0, 24, and 48 h after 30 days of germination. The samples were cleaned, frozen in liquid nitrogen, and stored at −80 °C until use.

### 4.2. Total RNA Extraction and cDNA Synthesis

Total RNA was extracted from different tissues (root, stem, leaf, and flower) of *A. vilmorinianum* using TRIzol reagent (Invitrogen, Waltham, MA, USA) and reverse-transcribed to cDNA, according to the manufacturer’s instructions with the PrimeScript™ II 1st Strand cDNA Synthesis Kit (Takara). The quality and quantity of the total RNA were verified by the ratio of OD_260_ and OD_280_ recorded with a UV-DS-11 spectrophotometer (DENOVIXINC, Wilmington, DE, USA) and gel electrophoresis.

### 4.3. Molecular Cloning of AvCPRs

The resulting cDNAs were used as templates for full-length cloning and qRT-PCR analysis. The primers are listed in Table S1. The open reading frame (ORF) of CPR genes was PCR amplified using Prime STAR HS (Premix) (Takara) and introduced into the expression vector pET-32a in the *Bam*H I and *Sal* I sites using the In-Fusion Snap Assembly Master Mix (Clontech). Positive results were verified via nucleotide sequencing at Sangon Biotech Co., Ltd. (Shanghai, China).

### 4.4. Bioinformatic Analysis of AvCPRs

The sequence alignment and homology analysis of AvCPRs were performed using the BLAST (http://www.ncbi.nlm.nih.gov/BLAST, accessed on 29 May 2020). The physicochemical properties, hydrophilicity levels, and transmembrane regions of the deduced proteins were predicted using the ExPASy ProtParam tool (http://web.expasy.org/protparam, accessed on 10 October 2020) and TMHMM 2.0 software (http://www.cbs.dtu.dk/services/TMHMM-2.0, accessed on 10 October 2020). The amino acid sequences of AvCPRs were aligned with reference sequences using DNAMAN V6.0.3.99 software (Lynnon Biosoft, San Ramon, CA, USA). The phylogenetic tree of CPRs was constructed using the MEGA 6.0 program with a bootstrap value of 1000.

### 4.5. Heterologous Expression of AvCPRs in E. coli

Transmembrane domain analysis showed that both AvCPR1 and AvCPR2 contained a transmembrane domain at the N-terminus. To increase protein solubility, the N-terminal membrane-anchoring domain of AvCPRs was truncated. The truncated AvCPRs were amplified and inserted into the expression vector pET-32a, yielding pET-32a-*AvCPRs*. The recombinant vectors were transformed into *Escherichia coli* BL21 (DE3) (TransGen Biotech, Beijing, China). For protein expression, a single colony of the recombinant strain was cultured in a Luria–Bertani (LB) medium containing 100 μg/mL of ampicillin and induced for 12 h with 1 mM isopropyl β-D-1-thiogalactopyranoside (IPTG, Biofroxx), as described [11]. The cells were collected via centrifugation at 4000× *g* for 15 min at 4 °C and washed with PBS buffer (pH 7.4) twice. Subsequently, the cells were collected and resuspended in the same buffer and disrupted by sonication for 5 s 10 times on ice. The lysate was centrifuged at 13,201× *g* for 20 min at 4 °C. The supernatant was loaded on a Ni-NTA agarose column and eluted with wash buffer (50 mM NaH_2_PO_4_, 300 mM NaCl, and 500 mM imidazole (pH 7.4)). Protein samples were separated using SDS-PAGE. The concentration of recombinant AvCPRs was quantified with an Enhanced BCA Protein Assay Kit (Beyotime, Shanghai, China). The ultraviolet absorption spectra of recombinant proteins in the range of 300–700 nm were measured in 100 mM Tris-HCl buffer containing 0.1 mM EDTA (pH 7.4) at 25 °C using Multiskan Go (Thermo Scientific, Vantaa, Finland).

### 4.6. Enzymatic Activity of AvCPRs In Vitro

The reactions for assaying AvCPR activities were carried out in 100 mM Tris-HCl buffer containing 0.1 mM EDTA (pH 7.4) at 25 °C. The reduction of cytochrome *c* was measured by the increase in absorbance at 550 nm. A molar absorption coefficient of 21 mM^−1^ cm^−1^ for cytochrome *c* was used for quantification. The reduction of potassium ferricyanide was monitored at 424 nm with a molar absorption coefficient of 1.02 mM^−1^ cm^−1^. To determine the kinetic parameters for cytochrome *c* or potassium ferricyanide, 4 μg of recombinant enzyme was added to various concentrations of cytochrome *c* (0–200 μM) or potassium ferricyanide (0–200 μM), and then a final concentration of 100 μmol/L NADPH was added to initiate the reaction. Ultraviolet absorption was recorded on a Multiskan Go (Thermo Scientific, Finland). The substrate concentration for half-maximal activity (*K_m_*) and the maximum rate of reaction (*V_max_*) were calculated using GraphPad Prism 5.

### 4.7. Heterologous Expression in Yeast

CPR is the reduction partner of P450s, affecting the catalytic activities of P450s. Since no P450s in *A. vilmorinianum* have been identified thus far, *CYP76AH1* in *Salvia miltiorrhiza* [40], which catalyzes miltiradiene to ferruginol, was utilized to confirm the interaction between *AvCPRs* and plant P450 in vivo. The ORFs of *SmCPR*1 or *AvCPR*s and *CYP76AH1* were inserted into the yeast expression vector pESC-His. The resulting recombinant plasmids pESC-His/pESC-His-*AH1*/pESC-His-*AH1*-SmCPR/pESC-His-*AH1-AvCPR1* and pESC-His-*AH1-AvCPR2* were expressed in the yeast strain YJ14 [20]. The yeast strain expressing pESC-His-*AH1*-*SmCPR* was used as a positive control. Yeasts harboring pESC-His-*AH1* or empty vector pESC-His were used as negative controls. All transformants were induced by 2% galactose for 48 h after culturing for 48 h in a synthetic complete medium without histidine and tryptophan (SD-His-Trp). The cell lysates were extracted with an equal volume of ethyl acetate. Samples were filtered through 0.22 μm filters and analyzed using a Waters 2695 HPLC system with a ZORBAX Extend-C_18_ column (4.6 × 250 mm, 5 μm). The column temperature was set at 30 °C. The flow rate was set at 1 mL/min. The mobile phases were water (A) and acetonitrile (B). The gradient was as follows: 0–20 min, 70–100% B; 20–25 min, 100% B; 25–27 min, 100–70% B; and 27–32 min, 70% B. The injection volume was 10 μL and the detection wavelength was 280 nm.

### 4.8. Expression Analysis of AvCPRs in A. vilmorinianum

The expression patterns of *AvCPR1* and *AvCPR2* in different organs (roots, stems, leaves, and flowers) of *A. vilmorinianum* and the leaves of MeJA-treated *A. vilmorinianum* were monitored via qRT-PCR. The *EF1α* gene of *A. vilmorinianum* was used as an internal control gene to normalize the expression value [41]. The primers for qRT-PCR analysis are listed in Table S1. The reactions were performed on a LightCycler^®^ 96 SW 1.1 (Roche, Indianapolis, IN, USA) with a SYBR Premix Ex Taq II system (Takara), according to the manufacturer’s instructions. The PCR conditions were as follows: preincubation at 95 °C for 30 s, 95 °C for 5 s, 60 °C for 30 s, and 72 °C for 10 s (40 cycles); melting at 95 °C for 15 s; and cooling at 65 °C for 30 s, 97 °C for 1 s, and 50 °C for 30 s. Calibration curves for each of the primer pairs and quantification were carried out using Roche LightCycler^®^ 96 SW 1.1 analysis software. Each reaction was repeated at least 3 times.

### 4.9. Homology Modeling and Molecular Docking

The modeling of the three-dimensional structures of AvCPR proteins was performed using the Phyre2 server. The crystal structure of ATR2 from *Arabidopsis thaliana* (PDB_ID: 5GXU) was used as the template. The structures of ligands were retrieved from the Protein Data Bank (PDB; http://www.rcsb.org/pdb/, accessed on 20 December 2022). The docking modeling was carried out with AutoDock Vina. The docking models and active binding sites were visualized in Chimera 1.15 and PyMol 2.0 software, respectively.

## Figures and Tables

**Table 1:** The steady-state kinetic constants of recombinant AvCPR proteins.

		*V_max_* (μmol·min^−1^·mg^−1^)	*K_m_* (μmol·L^−1^)	*K _cat_* (min^−1^)
Cytochrome *c*	AvCPR1	24.31 ± 3.55	344.20 ± 69.68	607.00 ± 88.75
	AvCPR2	17.44 ± 0.86	78.11 ± 9.15	174.4 ± 8.60
K_3_Fe(CN)_6_	AvCPR1	1.19 ± 0.11	35.68 ± 9.31	29.75 ± 2.75
	AvCPR2	1.37 ± 0.08	38.75 ± 5.57	13.70 ± 0.80

## Data Availability

All data generated or analyzed during this study are included in this published article and its supplementary information files.

## Supplementary Materials

The following supporting information can be downloaded at: https://www.mdpi.com/article/10.3390/molecules28217409/s1.
