# Supplementary material for: Cloning and Functional Characterization of NADPH-Cytochrome P450 Reductases in Aconitum vilmorinianum
Source: Molecules. 2023 Nov 3;28(21):7409. doi: 10.3390/molecules28217409 (PMC10648341; doi:10.3390/molecules28217409)

Cloning and Functional Characterization of NADPH-Cytochrome P450 Reductases in *Aconitum vilmorinianum*

Jingping Cheng [1], Guodong Li [1], Xue Wang [1], Congwei Yang [1], Furong Xu [1], Zigang Qian [1,*] and Xiaohui Ma [1,2,*]

1 College of Chinese Materia Medica, Yunnan Key Laboratory of Southern Medicinal Utilization, Yunnan University of Chinese Medicine, Kunming 650500, China
2 Key Laboratory of Yunnan Provincial Department of Education on Substance Benchmark Research of Ethnic Medicines, Kunming 650500, China
* Correspondence: qianzig@aliyun.com (Z.Q.); maxiaohui1988@126.com (X.M.)

Table S1. List of primers used in this study.

Primer code	Sequence
32a-AvCPR1-F	GGTACCCTCGAGGGATCCATGCAATCCGATTCGGTGAAAATC
32a-AvCPR1-R	TCTAGACTGCAGGTCGACTCACCATACATCACGGAGATACCGT
32a-AvCPR2-F	GGTACCCTCGAGGGATCCATGGACTCGGAGTCGGTGAAG
32a-AvCPR2-R	TCTAGACTGCAGGTCGACTTACCATACATCACGCAGATACCTT
32a-tAvCPR1-F	ATGGCTGATATCGGATCCATGAGGAGATCTTCCGCCAAG
32a-tAvCPR1-R	GCGGCCGCAAGCTTGTCGACGTCACCATACATCACGGAGATAC
32a-tAvCPR2-F	ATGGCTGATATCGGATCCATGAGAAGATCGTCGGGGAAG
32a-tAvCPR2-R	GCGGCCGCAAGCTTGTCGACGTTACCATACATCACGCAGATACC
His-AvCPR1-F	AAGAATTTTTGAAAATTCATGCAATCCGATTCGGTGAAA
His-AvCPR1-R	GTAATCCATCGATACTAGTCACCATACATCACGGAGATAC
His-AvCPR2-F	AAGAATTTTTGAAAATTCATGGACTCGGAGTCGGTGAAG
His-AvCPR2-R	GTAATCCATCGATACTAGTTACCATACATCACGCAGATACC
qPCR-AvCPR1-F	GGCTTCAGGACCTTCAGTTT
qPCR-AvCPR1-R	CACCATGTTCAGCAAGGATTTC
qPCR-AvCPR2-F	CGATCCTCTACGGAACTCAAAC
qPCR-AvCPR2-R	ACCACTTTGAATACTGCCTTCT

Table S2. Protein physical and chemical properties of AvCPRs.

Protein physical and chemical properties	AvCPR1	AvCPR2
Molecular weight	78.00 kDa	77.57 kDa
Theoretical pI	5.46	5.24
Formula	$C_{3481}H_{5419}N_{937}O_{1046}S_{27}$	$C_{3460}H_{5385}N_{919}O_{1051}S_{28}$
Instability index	44.09	35.78
Aliphatic index	80.97	80.67
Grand average of hydropathicity	-0.300	-0.268

Table S3. Secondary Structure of AvCPRs.

Secondary Structure	AvCPR1	AvCPR2
Alpha helix	285 (40.71%)	294 (42.06%)
Beta turn	32 (4.57%)	27 (3.86%)
Bend region	103 (14.71%)	97 (13.88%)
Random coil	280 (40.00%)	281 (40.20%)

Figure S1. Transmembrane region analyses of AvCPR proteins. **(a)** AvCPR1. **(b)** AvCPR2.

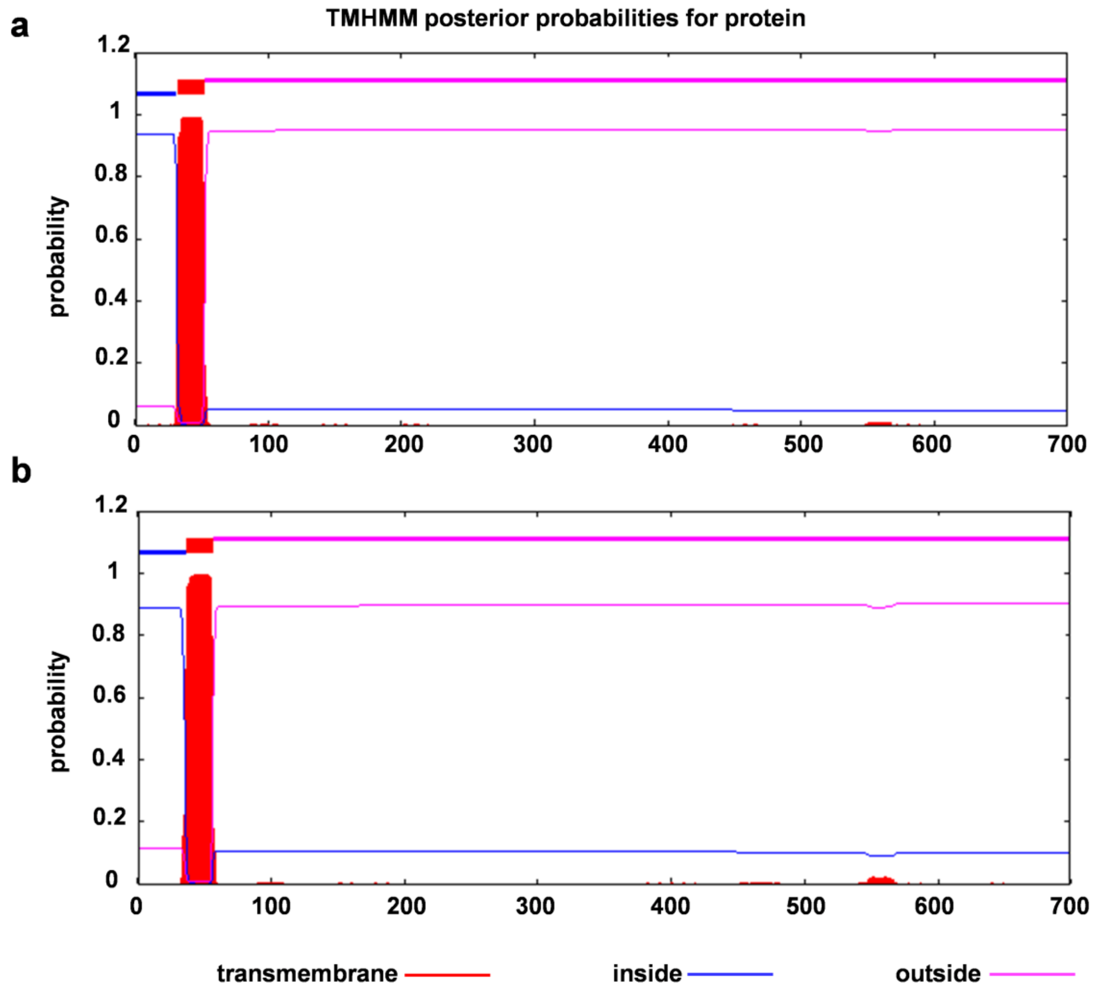

Figure S2. The predicted 3-D structure of AvCPRs. **(a, c)** AvCPR1. **(b, d)** AvCPR2. **(a-b)** 3-D structure of AvCPRs built by Phyre 2 server. **(c-d)** functional and structural residues of AvCPRs with high scores were highlighted in purple.

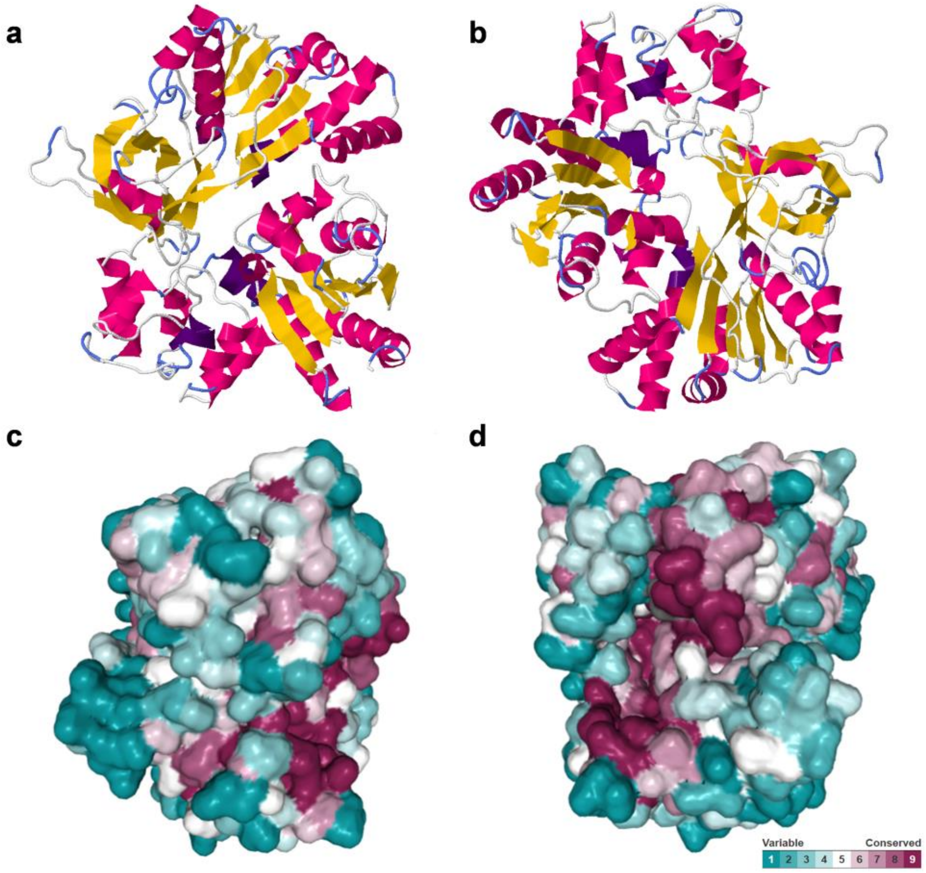

Figure S3. Spatial conformation of AvCPRs bound to ligands. The ligands FMN, FAD and NADPH are shown as blue, purple and light blue, respectively. The conformations were built on Chimera 1.15.

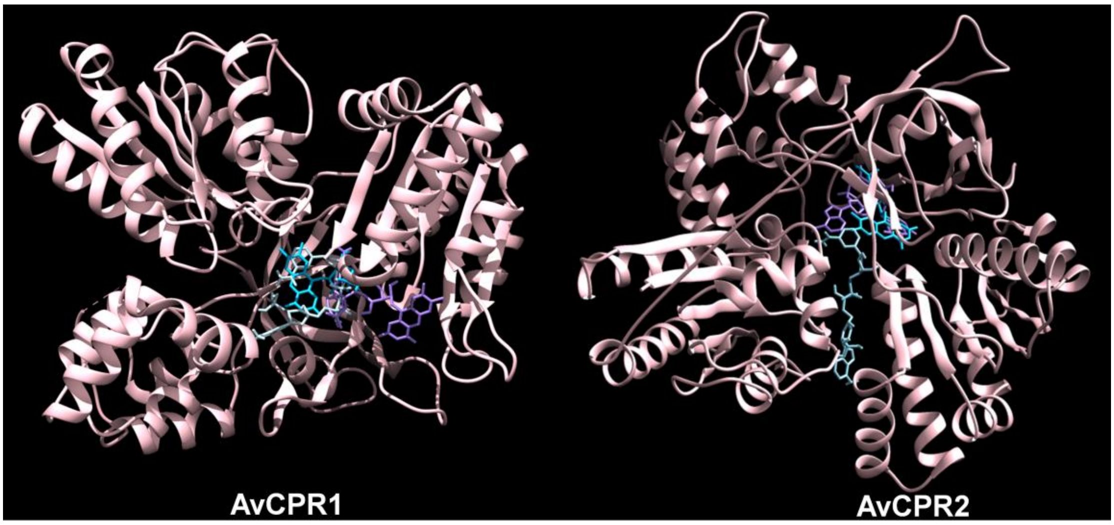

Figure S4. Electrostatic energy of AvCPRs binding surface to FMN and FAD. (a) AvCPR1. (b) AvCPR2. The ligands FMN and FAD are shown as blue and warm pink, respectively. The active binding sites were visualized in PyMol software.

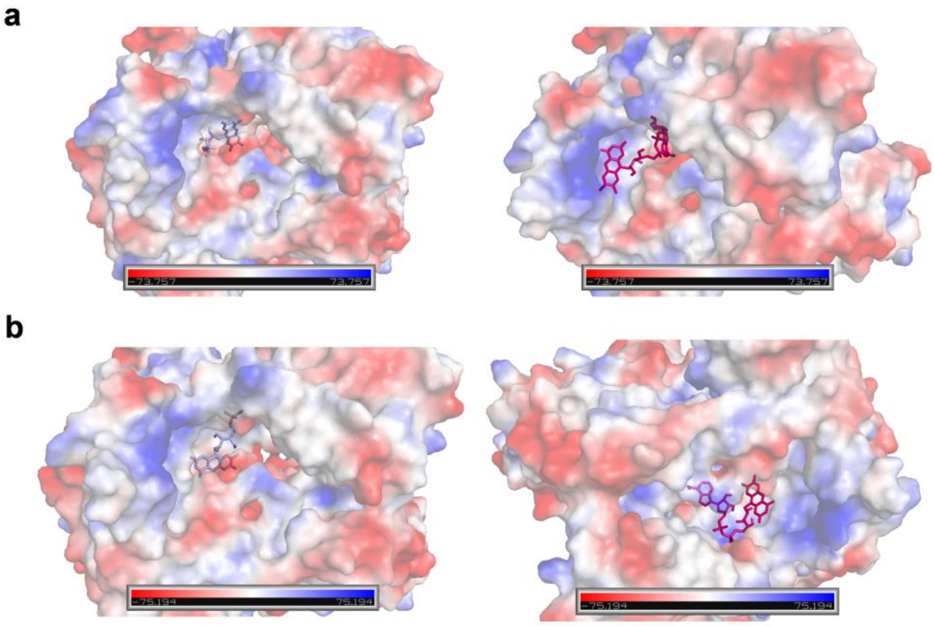

Supplement: Supplementary file 1 [file molecules-28-07409-s001.zip › molecules-2608360-supplementary.pdf]
